# Inhibition Lysosomal Degradation of Clusterin by Protein Kinase D3 Promotes Triple‐Negative Breast Cancer Tumor Growth

**DOI:** 10.1002/advs.202003205

**Published:** 2021-01-06

**Authors:** Yan Liu, Yehui Zhou, Xinxing Ma, Liming Chen

**Affiliations:** ^1^ The Key Laboratory of Bio‐Medical Diagnostics Suzhou Institute of Biomedical Engineering and Technology Chinese Academy of Sciences Suzhou 215163 P. R. China; ^2^ Cancer Institute Department of Biochemistry Jiangsu Key Laboratory for Molecular and Medical Biotechnology College of Life Science Nanjing Normal University Nanjing 210023 P. R. China; ^3^ The First Affiliated Hospital of Soochow University Soochow University Suzhou 215006 P. R. China

**Keywords:** clusterin, protein kinase D3, targeted therapies, triple‐negative breast cancer, tumor growth

## Abstract

Triple negative breast cancer (TNBC), with its lack of targeted therapies, shows the worst mortality rate among all breast cancer subtypes. Clusterin (CLU) is implicated to play important oncogenic roles in cancer via promoting various downstream oncogenic pathways. Here, protein kinase D3 (PRKD3) is defined to be a key regulator of CLU in promoting TNBC tumor growth. Mechanically, PRKD3 with kinase activity binding to CLU is critical for CLU protein stability via inhibiting CLU's lysosomal distribution and degradation. CLU and PRKD3 protein level are significantly elevated and positively correlated in collected TNBC tumor samples. CLU silencer (OGX‐011) and PRKDs inhibitor (CRT0066101) can both result in impressive tumor growth suppression in vitro and in vivo, suggesting targeting CLU and its key regulator‐PRKD3 are promisingly efficient against TNBC. Finally, secreted CLU (sCLU) is found to be elevated in serums from TNBC patients and reduced in serum from TNBC murine models post OGX‐011 and/or CRT0066101 treatment, suggesting serum sCLU is a promising blood‐based biomarker for clinical management of TNBC. Taken together, this study provides a thorough molecular basis as well as preclinical evidences for targeting CLU pathway as a new promising strategy against TNBC via revealing PRKD3 as the key regulator of CLU in TNBC.

## Graphical abstract

This study defines protein kinase D3 (PRKD3) to be the key regulator of clusterin (CLU) in promoting triple negative breast cancer (TNBC) tumor growth. PRKD3 inhibits CLU's lysosomal degradation. A remarkable therapeutic effect is achieved against CLU pathway via targeting CLU and PRKD3 for treatment of TNBC. Secreted CLU in serum is a blood‐based biomarker for diagnosis of CLU+ TNBC.

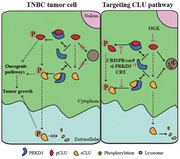

## 1 Introduction

Breast cancer is the most common cancer type in women.^[^ ^1^ ^]^ Triple‐negative breast cancer (TNBC) lacks expression of estrogen receptor (ER), progesterone receptor, and human epidermal growth factor receptor 2 (HER2).^[^ ^2^ ^]^ Unlike ER+ and HER2+ breast cancer subtypes, due to lack of druggable discriminators and established targeted therapies for TNBC, patients with TNBC can only receive non‐specific treatments, such as surgery, chemotherapy, and radiotherapy.^[^ ^3^ ^]^ To date, TNBC still shows the worst mortality rate among all breast cancer subtypes.^[^ ^4^ ^]^ Therefore, it remains an urgent and unmet clinical need for further exploring the pathological mechanism of TNBC to identify new drivers in TNBC as discriminators and therapeutic targets to improve clinical management of patients with TNBC.

Clusterin (CLU) is documented to be a stress‐activated, ATP‐independent molecular chaperone, and its mature form is normally secreted from cells.^[^ ^5^ ^]^ Studies have implicated that CLU play important oncogenic roles in many cancers.^[^ ^6^, ^7^ ^]^ CLU is reported to be up‐regulated in breast cancers^[^ ^6^, ^8^ ^]^ and other cancers, including prostate,^[^ ^9^, ^10^, ^11^, ^12^ ^]^ gastric,^[^ ^13^, ^14^ ^]^ pancreatic,^[^ ^15^ ^]^ colon,^[^ ^16^, ^17^ ^]^ and head and neck.^[^ ^18^ ^]^ Previous studies have revealed many CLU's downstream targets in cancer, including those for inhibition of cell death pathways and modulation of pro‐survival signaling to promote cell growth.^[^ ^6^ ^]^ However, regulators of CLU in pathogenesis of cancer are waiting to be explored.

Protein kinase D3 (PRKD3) belongs to the multigene protein kinase D family of serine/threonine kinases, which has been implicated to regulate cancer progression in a broad range of cancer types.^[^ ^19^, ^20^ ^]^ Our groups have identified PRKD3 as a breast cancer susceptibility gene using transposon insertional mutagenesis in mice, implicating a potential important oncogenic role of PRKD3 in breast cancer.^[^ ^21^ ^]^ In this study, we define PRKD3 to be a key regulator of CLU. PRKD3 directly binds and stabilizes CLU to promote TNBC tumor growth. CLU protein stability requires PRKD3, which stabilize CLU via inhibiting CLU's lysosomal not proteasomal degradation. Loss of PRKD3's kinase activity accumulates CLU distribution to lysosomes for lysosomal degradation as loss of PRKD3 protein does, suggesting that CLU stabilized by PRKD3 involves phosphorylation modifications. CLU stabilized by PRKD3 is critical for promoting TNBC tumor cell growth in vitro and in vivo. In our collected tumor samples from TNBC patients, CLU and PRKD3 protein level are found to be significantly positively correlated, supporting the positive regulatory relation between CLU and PRKD3. More than 80% collected TNBC tumor samples show to be CLU positive (CLU+), suggesting CLU+ TNBC represents the most common TNBC type. Importantly, we provide both in vitro and in vivo preclinical experimental evidences to show the efficiency of CLU silencer (OGX‐011) and PRKDs inhibitor (CRT0066101) treatment in TNBC tumor growth suppression, suggesting targeting CLU pathway will be promisingly efficient for treatment of TNBC. Finally, elevated secreted CLU (sCLU) was found in serums from TNBC patients compared to healthy controls, OGX‐011 and/or CRT0066101 treatment resulted in reduced sCLU in serum from TNBC murine models with inhibition of TNBC tumor growth, suggesting potential application of serum sCLU as a blood‐based biomarker for clinical management of TNBC. Thus, this study reveals a new mechanism of CLU pathway in TNBC via defining PRKD3 as a new key regulator of CLU, and provides new strategies for clinical management of TNBC via raising CLU and PRKD3 as two promising therapeutic targets and serum sCLU as a blood‐based biomarker.

## 2 Results

### 2.1 Physical Interaction between CLU and PRKD3

**Figure 1:**
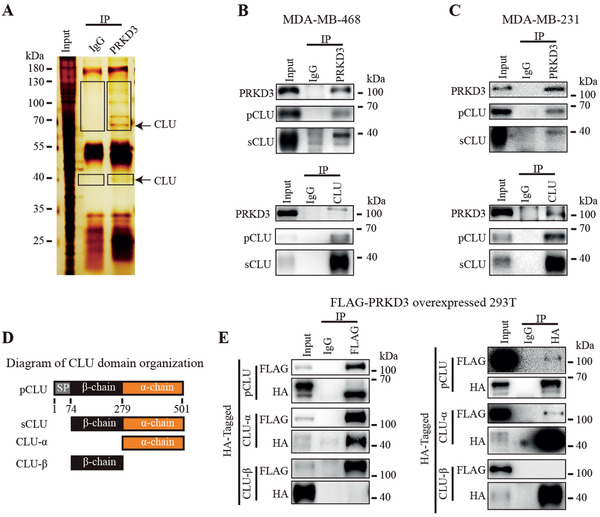
PRKD3 binds CLU protein. A) Representative silver‐staining gel shows whole protein lysis as input and proteins pulled‐down by IgG and anti‐PRKD3 antibody. Box‐marked regions indicate the protein bands specifically presented in anti‐PRKD3 antibody pulled‐down sample compared to IgG control sample, and were subjected to identification by MS. B,C) Representative western blot images from Co‐IP assays show that PRKD3 binds CLU in two TNBC cell lines: B) MDA‐MB‐468 and C) MDA‐MB‐231. D) A schematic diagram shows the domain organization of protein coded by CLU gene. SP: Signal Peptide. E) Representative western blot shows that over‐expressed FLAG‐PRKD3 interacts with overexpressed HA‐pCLU, HA‐CLU‐*α* but not HA‐CLU‐*β* in 293T.

Our previous study identified PRKD3 as a candidate breast cancer driver gene.^[^ ^21^ ^]^ When we investigated the PRKD3 interactome using co‐immunoprecipitation (Co‐IP) followed by mass spectrum (MS) identification, we found CLU specifically in the anti‐PRKD3 antibody pull‐down sample, but not in the control IgG sample (**Figure** **1**A and Table S1, Supporting Information). During biogenesis of CLU, CLU precursor (pCLU ≈60 kDa) are maturated to be secreted CLU (sCLU containing *α* and *β* chain of ≈40 kDa).^[^ ^22^ ^]^ According to this information, CLU is likely to be presented in the two strong bands near the molecular markers of 70 and 40 kDa specifically in the anti‐PRKD3 antibody pull‐down sample, but not in the control IgG sample (Figure 1A). Then, we used two TNBC cell lines (i.e., MDA‐MB‐468 and MDA‐MB‐231) to validate the physical interaction between endogenous CLU and PRKD3 via Co‐IP using both anti‐CLU and anti‐PRKD3 antibody. The results show that anti‐PRKD3 antibody can pull down both pCLU and sCLU, and anti‐CLU antibody can pull down PRKD3, confirming the physical interaction between CLU and PRKD3 (Figure 1B,C). Domain analysis on pCLU reveals that pCLU contains a signaling peptide followed by *α*‐ and *β*‐chain domain (Figure 1D). To inspect the essential domain in CLU for the interaction between CLU and PRKD3, we overexpressed HA‐tagged CLU or its serial domain truncation mutants in 293T with ectopic overexpression of FLAG‐tagged PRKD3 and carried out Co‐IP using anti‐HA and anti‐FLAG antibody. The results show that CLU *α*‐chain domain but not *β*‐chain domain is responsible for the interaction between CLU and PRKD3 (Figure 1E).

### 2.2 CLU Protein Not Its mRNA Level Is Positively Regulated by PRKD3

**Figure 2:**
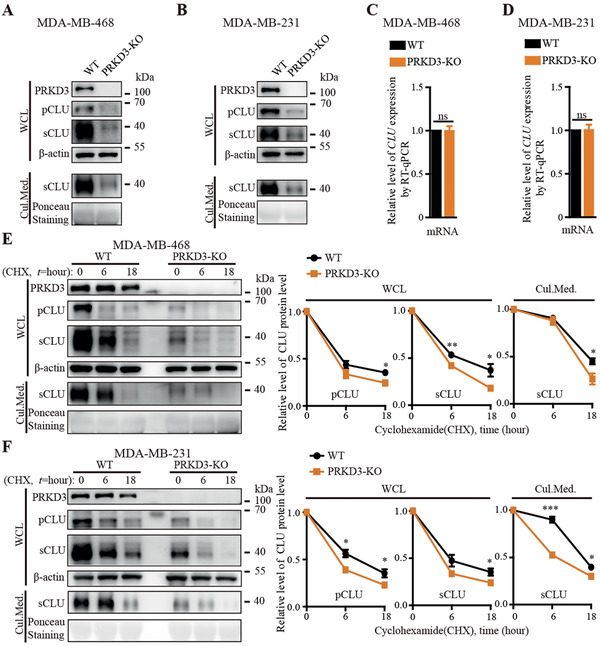
PRKD3 stabilizes CLU protein. A,B) Representative western blot shows that knock out PRKD3 (PRKD3‐KO) in TNBC cells resulted decrease of protein levels of pCLU and sCLU in cells as well as sCLU in cell culture medium in two PRKD3‐KO TNBC cell lines compared to their parental WT cells: A) MDA‐MB‐468 and B) MDA‐MB‐231. C,D) RT‐qPCR results show that there are no changes of mRNA levels of CLU in two PRKD3‐KO TNBC cell lines compared to their parental WT cells: C) MDA‐MB‐468 and D) MDA‐MB‐231. E,F) Representative western blots for CHX‐chasing assays show that there are consistent decreases of protein stabilities of pCLU and sCLU in cells as well as sCLU in cell culture mediums in two PRKD3‐KO TNBC cell lines compared to their parental WT cells: E) MDA‐MB‐468 and F) MDA‐MB‐231. The error bars represent the mean ± SEM. Statistics analyses were performed using *t*‐test: *p < 0.05, **p < 0.01, and ***p < 0.001.

The regulators of CLU in TNBC are largely unknown. To further test whether and how PRKD3 functions as a regulator of CLU via directly binding, we examined the CLU protein level using western blot in TNBC cells with PRKD3 depleted through knockout PRKD3 (PRKD3‐KO) using CRISPR/Cas9 and knockdown PRKD3 using anti‐PRKD3 siRNAs compared to controls. In both cases, we consistently observed a sharp decrease in protein level of CLU (pCLU, sCLU) from cell lysates as well as sCLU in the cell culture medium upon depletion PRKD3 compared to controls, suggesting that PRKD3 positively regulates CLU protein level (**Figure** **2**A,B and Figure S1A–F, Supporting Information). To test whether PRKD3 positively regulated CLU expression level via modulating the transcription of CLU gene, we examined the CLU mRNA upon PRKD3 depletion compared to controls. The results show that depletion PRKD3 is unable to affect mRNA expression of CLU, suggesting that PRKD3 doesn't regulate CLU gene transcription (Figure 2C,D, and Figure S1G,H, Supporting Information). We then hypothesized that PRKD3 regulates CLU protein level by affecting its protein stability. To test this idea, we used a protein synthesis inhibitor‐cycloheximide (CHX) to treat TNBC cells, and found that depletion PRKD3 in TNBC cells consistently reduced the stability of CLU (pCLU, sCLU) in cells as well as sCLU in the cultural medium (Figure 2E,F and Figure S1I–L, Supporting Information). These results suggest that PRKD3 is critical for CLU protein stability.

### 2.3 PRKD3 Inhibits CLU's Lysosomal Distribution and Degradation

**Figure 3:**
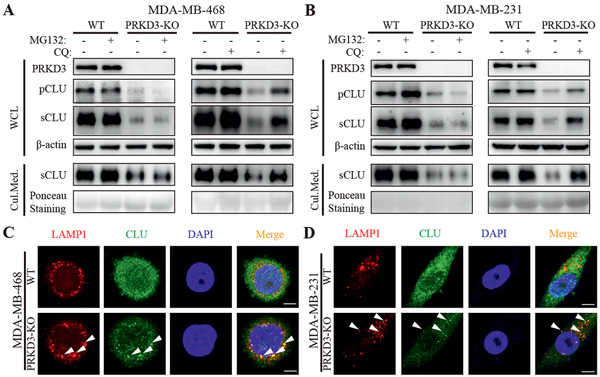
PRKD3 inhibits degradation of CLU via inhibiting lysosomal pathway not proteasomal pathway. A,B) Representative western blots consistently show that treatment of lysosomal degradation inhibitor‐CQ but not proteasomal degradation inhibitor‐MG132 can increase protein levels of pCLU and sCLU in cells as well as sCLU in cell culture medium in two PRKD3‐KO TNBC cell lines compared to WT cells: A) MDA‐MB‐468 and B) MDA‐MB‐231. C,D) Representative confocal images from immunofluorescent assay using LAMP1 as the lysosome marker consistently show that there are obvious increases of CLU‐LAMP1 colocalization foci in two PRKD3‐KO TNBC cell lines compared to WT cells: C) MDA‐MB‐468 and D) MDA‐MB‐231. Several representative CLU‐LAMP1 colocalization foci were indicated by arrows. Scale bar indicates 2.5 µm.

Then, we further investigated how PRKD3 stabilizes CLU protein. There are two major protein degradation systems in cells to regulate proteins’ stabilities: ubiquitin‐proteasome system (UPS)^[^ ^23^, ^24^ ^]^ and autophagy‐lysosome pathway (ALP).^[^ ^25^ ^]^ To test whether PRKD3 stabilizes CLU protein through either UPS or ALP or both, proteasomal inhibitor‐MG132 and lysosomal inhibitor‐chloroquine (CQ) were used. The results show that CQ but not MG132 treatment can alleviate the degradation of CLU upon depletion of PRKD3, suggesting that PRKD3 stabilizes CLU via protecting CLU from lysosomal degradation (**Figure** **3**A,B and Figure S2A–F, Supporting Information). LAMP1 is a documented lysosome marker.^[^ ^26^, ^27^, ^28^ ^]^ To further test PRKD3 inhibits lysosomal degradation of CLU via inhibiting CLU transported to lysosomes, using LAMP1 anti‐bodies to label lysosomes, in immune‐fluorescence assay, we found that depletion PRKD3 increased the distribution of CLU to lysosomes compared to controls (Figure 3C,D and Figure S2G–L, Supporting Information). Previous studies have shown that low density lipoprotein‐related protein 2 (LRP2) is an endocytic receptor that internalizes CLU directed to the lysosomal degradation pathway.^[^ ^29^, ^30^ ^]^ We hypnotized that PRKD3 binds to CLU to inhibit LRP2‐mediated lysosomal distribution and degradation of CLU. To test this idea, we carried out Co‐IP using anti‐CLU antibodies in PRKD3‐KO cells compared to WT cells. As anticipated, the results show that protein level of LRP2 pulled‐down by CLU antibody is significantly increased in PRKD3‐KO cells compared to control WT cells (Figure S2M–P, Supporting Information). These findings strongly suggest that PRKD3 stabilizes CLU via inhibiting lysosomal distribution and degradation of CLU in LRP2‐mediated manner.

### 2.4 Kinase Activity of PRKD3 is Required for Stabilizing CLU

**Figure 4:**
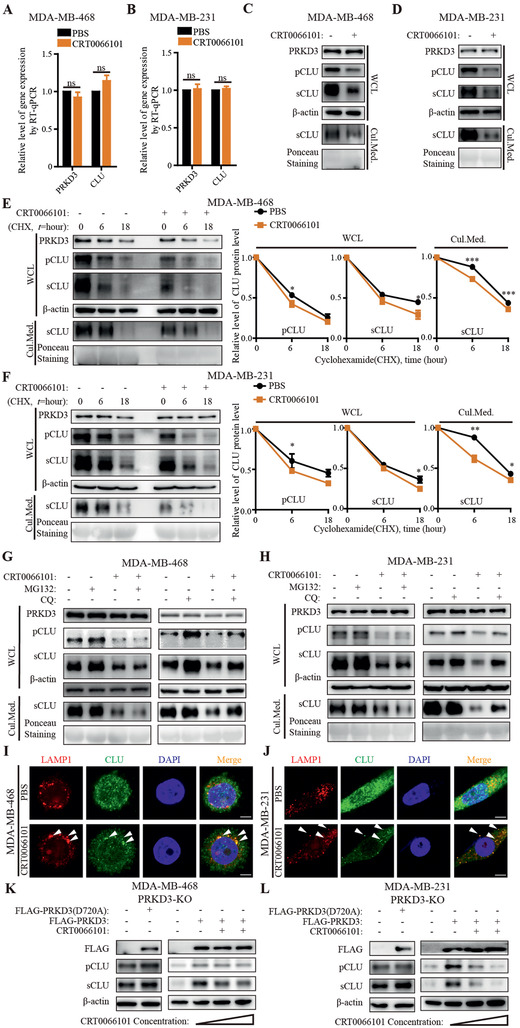
PRKD3 inhibits lysosomal degradation of CLU dependent on its kinase activity. A,B) RT‐qPCR results show that there are consistent no changes of mRNA levels of CLU upon treatment of PRKD3 kinase activity inhibitor‐CRT0066101 compared to PBS in A) MDA‐MB‐468 and B) MDA‐MB‐231. C,D) Representative western blots show that there are consistent decreases of protein levels of pCLU and sCLU in cells as well as sCLU in cell culture mediums upon treatment of CRT0066101 compared to PBS in C) MDA‐MB‐468 and D) MDA‐MB‐231. E,F) Representative western blots for CHX‐chasing assays show that there are consistent decreases of protein stabilities of pCLU and sCLU in cells as well as sCLU in cell culture mediums upon treatment of CRT0066101 compared to PBS in E) MDA‐MB‐468 and F) MDA‐MB‐231. G,H) Representative western blots consistently show that treatment of lysosomal inhibitor‐CQ but not proteasomal inhibitor‐MG132 can increase protein levels of pCLU and sCLU in cells as well as sCLU in cell culture mediums in G) MDA‐MB‐468 and H) MDA‐MB‐231 upon CRT0066101 treatment compared to PBS. I,J) Representative confocal images from immunofluorescent assays using LAMP1 as the lysosome marker consistently show that there are obvious increases of CLU‐LAMP1 colocalization foci in I) MDA‐MB‐468 and J) MDA‐MB‐231 upon treatment of CRT0066101 compared to PBS. Several representative CLU‐LAMP1 colocalization foci were indicated by arrows. Scale bar indicates 2.5 µm. K,L) Representative western blots consistently show that re‐expression of WT FLAG‐PRKD3 but not kinase‐dead mutant FLAG‐PRKD3 (D720A) can restored the CLU protein level in K) PRKD3‐KO MDA‐MB‐468 and L) MDA‐MB‐231, which can be re‐decreased upon CRT0066101 treatment. The error bars represent the mean ± SEM. Statistics analyses were performed using *t*‐test: *p < 0.05, **p < 0.01, and ***p < 0.001.

PRKD3 is a serine/threonine kinase, which exerts its biological function through its kinase activity.^[^ ^31^ ^]^ Then, we further tested whether PRKD3 inhibits CLU lysosomal degradation dependent on its kinase activity. Due to lack of PRKD3 specific inhibitor, we used a pan‐PRKD inhibitor CRT0066101.^[^ ^32^, ^33^, ^34^ ^]^ The results show that inhibiting PRKD3 kinase activity by CRT0066101 can reduce the CLU (pCLU, sCLU) protein level but not CLU mRNA level as depleting PRKD3 did in TNBC cells (Figures 2A–D, and **4**A–D; Figures S1A–H and S3A–D, Supporting Information). Furthermore, CRT0066101 treatment resulted in reduction of CLU protein stability and increase of CLU lysosomal distribution comparable to depletion PRKD3 did (Figures 2E,F, 3A–D, and 4E–J; Figures S2A–L and S3E–H, Supporting Information). To further confirm the specificity of PRKD3 kinase activity on stabilizing CLU protein, we re‐expressed wild type (WT) PRKD3 and kinase‐dead PRKD3 mutant (PRKD3^D720A^) in PRKD3‐KO TNBC cells. The results show that re‐expression of WT PRKD3 but not PRKD3^D720A^ can restore CLU protein level, which is re‐decreased in additional of CRT0066101 treatment (Figure 4K,L, Figure S3I,J, Supporting Information). Taken together, our data suggest that PRKD3 inhibits CLU lysosomal degradation dependent on its kinase activity and involved PRKD3‐mediated phosphorylation modifications.

### 2.5 CLU Stabilized by PRKD3 Promotes TNBC Tumor Growth In Vitro and In Vivo

**Figure 5:**
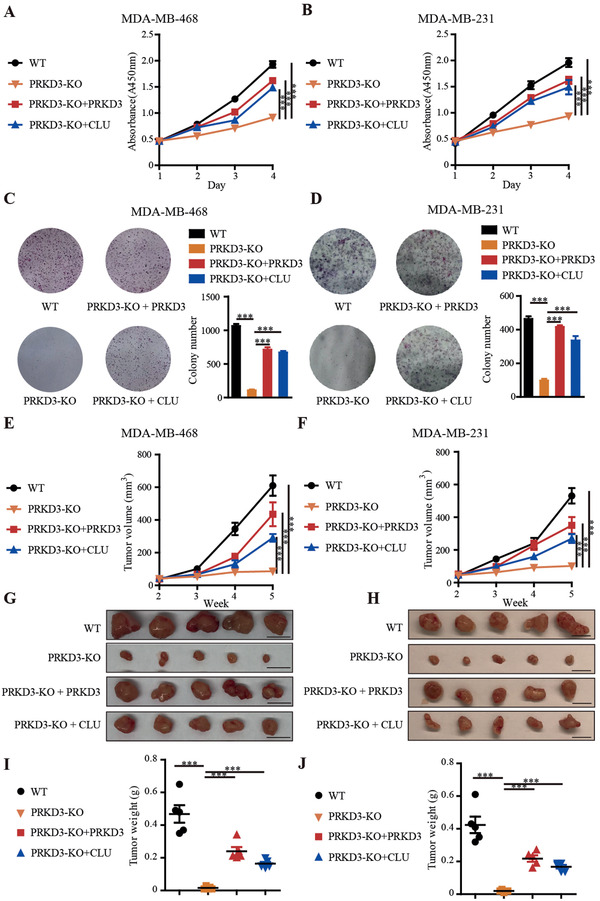
PRKD3 stabilizes CLU to promote TNBC tumor growth. A,B) Proliferation and C,D) colony formation assay consistently show that PRKD3‐KO cells exhibited reduced cell growth abilities in two TNBC cell lines compared to their parental WT cells, including A,C) MDA‐MB‐468 and B,D) MDA‐MB‐231, and either over‐expression of PRKD3 or CLU in PRKD3‐KO cells can restore the cell growth abilities. E–J) Results from xenograft tumor growth assays consistently show the reduced tumor growth in PRKD3‐KO compared to E,G,I) WT MDA‐MB‐468 and F,H,J) MDA‐MB‐231, and either over‐expression of PRKD3 or CLU in PRKD3‐KO cells can restore the tumor growth. Each group contains five mice (*n* = 5). The error bars represent the mean ± SEM. Statistics analyses were performed using *t*‐test: ***p < 0.001.

To further test the function of CLU stabilized by PRKD3 in pathogenesis of TNBC. First, we carried in vitro cell growth assays, including cell viability assay and colony formation assay. The results consistently show that knock out PRKD3 in TNBC cells reduced cell growth, and the related phenotypes can be rescued via either ectopic (over)expression of CLU or PRKD3 in PRKD3‐KO TNBC cells (**Figure** **5**A–D). Then, we further confirmed these findings in xenograft murine models using TNBC cancer cell lines. Consistent with in vitro cell assays, in mice, tumors formed by PRKD3‐KO TNBC cells show significantly reduced tumor volumes and weights compared to those formed by parental cells, and either ectopic (over)expression of CLU or PRKD3 in PRKD3‐KO TNBC cells can restore TNBC tumor growth (Figure 5E–J). Taken together, our data suggest that CLU stabilized by PRKD3 is required for TNBC tumor growth.

### 2.6 Clinical Relevance of CLU Stabilized by PRKD3 in TNBC

**Figure 6:**
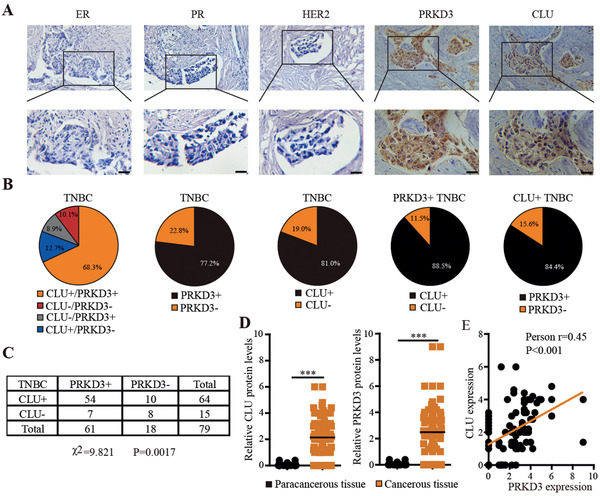
Clinical relevance of CLU stabilized by PRKD3. A) Representative Immunohistochemistry (IHC) images show that there is elevated expression of PRKD3 and CLU in TNBC tumors. Scale bar indicates 100 µm. B) Pie charts show that the dominant proportion of collected TNBC tumors have elevated PRKD3 expression (PRKD3+) and elevated CLU expression (CLU+). C) Summary of IHC analysis shows that there is a significantly positive co‐relationship between positivity of PRKD3 and CLU in TNBC tumors. D) Semi‐quantification the IHC analysis confirms the elevated expression of PRKD3 and CLU in TNBC cancer tissues compared to precancerous tissues. E) Co‐relationship analysis confirms the positive correlation between the expression of PRKD3 and CLU in TNBC tumors. The error bars represent the mean ± SEM. Statistics analyses were performed using *t*‐test: ***p < 0.001.

To further test the importance and reality of CLU stabilized by PRKD3 in TNBC, we further investigated clinical relevance of CLU and PRKD3 in our collected TNBC tumors from patients. First, we examined CLU and PRKD3 protein expression level in clinical TNBC tumor samples from patients by immunohistochemical analysis. Strikingly, we found that up to 90% TNBC samples are CLU+ and/or PRKD3‐positive (PRKD3+), while CLU and PRKD3 double negative (CLU−/PRKD3−) TNBC only accounts for ≈10% TNBC (**Figure** **6**A,B and Table S2, Supporting Information). In TNBC samples, more than 80% (81%) and about 80% (77.2%) TNBC tumor samples are CLU+ and PRKD3+, respectively (Figure 6B). And 84.4% CLU+ TNBC and 88.5% PRKD3+ TNBC is found to be also PRKD3+ and CLU+, respectively (Figure 6B). Around 70% (68%) TNBC samples are CLU+/PRKD3+ (Figure 6B). The positivity of CLU and PRKD3 in TNBC is found to be significantly correlated in clinical TNBC tumor samples (Figure 6C). Consistently, when CLU and PRKD3 protein expression level in TNBC sample were quantified and analyzed, both CLU and PRKD3 protein levels are significantly elevated in TNBC cancer tissues compared to paracancerous tissues, and are significantly positively correlated (Figure 6D,E). These data further support the positive regulation effect of PRKD3 on CLU in TNBC.

### 2.7 Efficiency of Targeted Therapies against CLU Pathway via Targeting CLU and PRKD3

**Figure 7:**
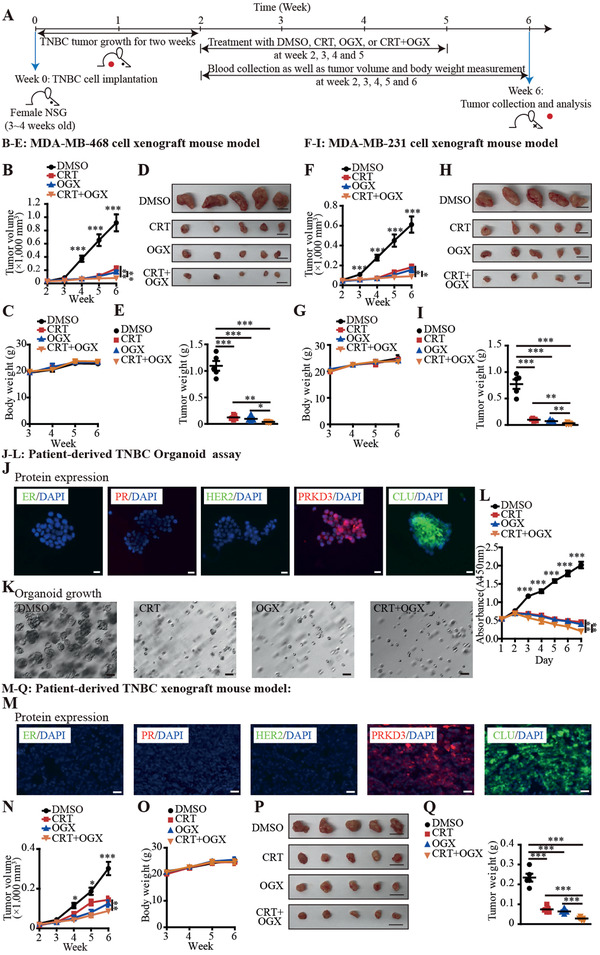
Efficiency of targeted therapies against CLU pathway via targeting CLU and/or its key regulator PRKD3 for treatment of TNBC. A) A schematic diagram deciphering the experiment flow for investigating the efficiency of targeted therapies against CLU pathway via targeting CLU using OGX‐011 (OGX) and/or PRKD3 using CRT0066101 (CRT) in treatment of TNBC. B–I) Results from xenograft mouse models using TNBC cell lines show the promising efficiency of targeted therapies against CLU pathway using OGX and/or CRT in suppressing TNBC tumor growth. Treatments by OGX and/or CRT can result in significantly growth suppressions without affecting body weights of mice with TNBC tumors formed by implantation of B–E) MDA‐MB‐468 and F–I) MDA‐MB‐231. B,F) Tumor growth across indicated time points. C,G) Body weights of mice across indicated time points. D,H) The collected tumors from mice. Scale bar indicates 1 cm. E,I) The weights of collected tumors from mice. Each group contains five mice (*n* = 5). J–L) Results from patient‐derived TNBC organoid assays show the efficiency of targeted therapies against CLU pathway using OGX and CRT. J) Reprehensive images confirm that TNBC tumors show lack expression of ER, PR, and HER2, and elevated expression of PRKD3 and CLU in the organoids. K) Reprehensive images show the reduced growth of the TNBC organoids upon treatment of OGX and/or CRT. L) Quantification analysis shows the efficient suppression of TNBC organoids’ growth by OGX and/or CRT. M–Q) Results from PDX TNBC mouse models show the promising efficiency of targeted therapies against PRKD3‐CLU axis. M) Reprehensive images confirm lack expression of ER, PR, and HER2, and elevated expression PRKD3 and CLU in PDX TNBC tumors. N) PDX tumor growth across indicated time points. O) Body weights of PDX mice across indicated time points. P) The collected tumors from PDX mice. Scale bar indicates 1 cm. Q) The weights of collected tumors from PDX mice. Scale bar indicates 50 µm. Each group contains five mice (*n* = 5). The error bars represent the mean ± SEM. Statistics analyses were performed using *t*‐test: *p < 0.05, **p < 0.01, and ***p < 0.001.

Above, we have defined the key role of PRKD3 for stabilizing CLU protein in TNBC. It remains an urgent and unmet clinical need for new targeted therapies to improve clinical management of patients with TNBC. Then, we further test whether targeted therapies against CLU pathway via targeting CLU and its regulator‐PRKD3 would be efficient for treatment of TNBC (**Figure** **7**A). Custirsen (OGX‐011) is an established antisense oligonucleotide drug which silences CLU expression.^[^ ^35^ ^]^ Due to lack of specific PRKD3 inhibitor, here, we used PRKDs inhibitor (CRT0066101). First, we tested efficiency of OGX‐011 (OGX) and CRT0066101 (CRT) against TNBC tumor growth in TNBC cancer cell line xenograft murine models (Figure 7B–I). The results show that systematic administration of OGX and CRT, alone and in combination, without affecting weight of mice, resulted in significant TNBC tumor growth suppression at ≈90%, ≈80%, and ≈95%, respectively (Figure 7B–I).

To further confirm the efficiency of targeted therapy against CLU pathway for treatment of TNBC, we generated patient‐derived TNBC organoid assays (Figure 7J–L) and patient‐derived xenograft (PDX) TNBC tumor murine models (Figure 7M–Q). Consistently, in both cases, OGX and CRT, alone and in combination, significantly suppress TNBC tumor growth. In PDX models, without affecting weight of mice, OGX and CRT, alone and in combination, results in TNBC tumor growth suppression at ≈70%, ≈65%, and ≈85%, respectively (Figure 7N–Q). These data suggest that targeted therapies against CLU pathway via targeting CLU and PRKD3 are promisingly efficient for treatment of TNBC.

### 2.8 Serum sCLU Provides a Blood‐Based Biomarker for Management of TNBC

**Figure 8:**
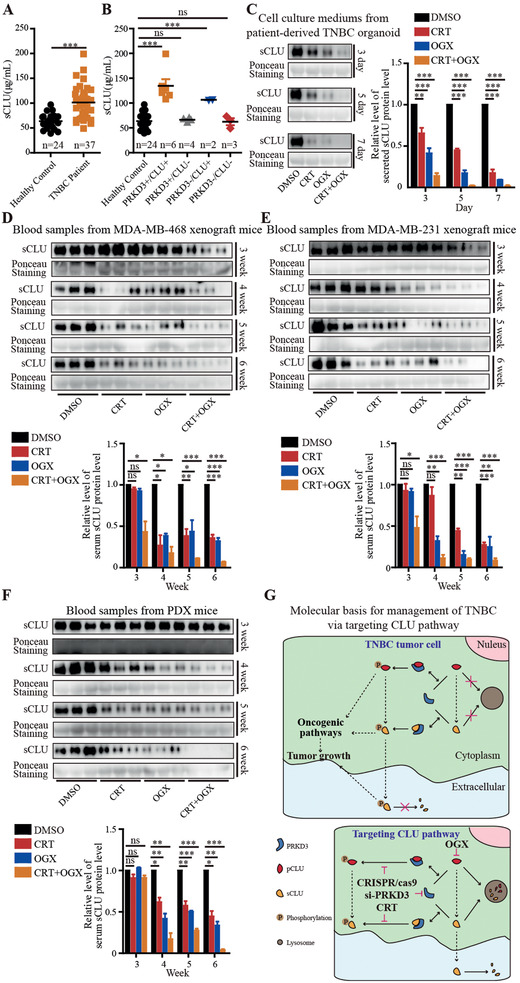
Serum sCLU provides a blood‐based biomarker for management of TNBC. A) Analysis of serum sCLU protein levels shows that TNBC patients have elevated serum sCLU compared to healthy controls. B) Blood samples from patients with CLU+ TNBC show highest serum sCLU level compared to other groups. C–F) Reprehensive western blots show decrease of secreted sCLU protein level upon treatment by OGX and/or CRT. C) Secreted sCLU in culture mediums from patient‐derived TNBC organoids. D–F) Serum sCLU in TNBC murine models treated with OGX and/or CRT compared to control. Serum sCLU in blood samples from D) MDA‐MB‐468 xenograft mice, E) MDA‐MB‐231 xenograft mice, and F) PDX mice. G) A proposed working model. The error bars represent the mean ± SEM. Statistics analyses were performed using *t*‐test: *p < 0.05, **p < 0.01, and ***p < 0.001.

The use of blood‐based biomarkers (“liquid biopsies”) can enable earlier diagnosis, lower costs by avoiding complex invasive procedures, tailoring molecular targeted treatments, improving patient convenience, and ultimately supplement clinical oncologic decision‐making, without given multiple limitations of obtaining tissue samples.^[^ ^36^, ^37^ ^]^ Maturation form of CLU, that is, sCLU, is normally secreted from cells.^[^ ^38^, ^39^ ^]^ In above, we have shown that secreted sCLU from cells is also controlled by PRKD3 (Figures 1, 2, 3, 4 and Figures S1–S3, Supporting Information). Then, we intended to further investigate whether sCLU in serum can be used as a biomarker for diagnosis of TNBC via collecting blood samples from both healthy persons and patients with TNBC. We found significantly elevated sCLU in serum from TNBC patients compared to healthy controls (**Figure** **8**A). Furthermore, when we inspected the CLU status in TNBC tumors from randomly selected 15 TNBC patients, we found that the protein level of sCLU in serum from patients with CLU+ TNBC is significantly higher than healthy controls (Figure 8B). These results together suggest that sCLU in serum is associated with the status of CLU in TNBC tumor, and can be used as a blood‐based biomarker for diagnosis of CLU+ TNBC, which represents the most common TNBC type. Then, we test whether sCLU can predict the response TNBC tumor upon treatment by targeted therapies against CLU pathway. When we examined the protein level in the cell culture mediums from TNBC organoid assays and murine blood samples from TNBC murine models upon treatment of the targeted therapies against CLU pathway via targeting CLU and/or PRKD3 compared to controls, we found that sCLU protein levels were consistently decreased upon treatment of the targeted therapies against CLU and PRKD3, which resulted in significant tumor suppression (Figure 8C–F). These results suggest that sCLU in serum can be used as a blood‐based biomarker applied to predict both the CLU+ TNBC tumor and the clinical outcome of TNBC with the targeted therapies against CLU pathway.

## 3 Discussion

CLU is implicated in a number of biological processes, including cell multiplication, cell survival, epithelial mesenchymal transition, metastasis, and mitosis.^[^ ^40^ ^]^ Previous studies have revealed many downstream oncogenic factors of CLU for its oncogenic roles in cancer progression via promoting various oncogenic pathways. For examples, CLU facilitates stress‐induced lipidation of LC3 and autophagosome biogenesis pathway to enhance cancer cell survival,^[^ ^6^ ^]^ regulates Twist1 to mediate TGF‐*β* pathway to induce epithelial mesenchymal transition and metastasis,^[^ ^40^ ^]^ and inhibits a constitutive activation of Cdc25C via the phosphatase PP2A to regulate Wee1–Cdk1 pathway to promote survival in cancer cells.^[^ ^10^ ^]^ However, regulators of CLU in pathogenesis of TNBC are largely unknown. In this study, we defined PRKD3 as a key regulator of CLU via inhibiting lysosomal degradation of CLU to promote TNBC tumor growth.

Breast cancer is the most common cancer in women.^[^ ^41^, ^42^, ^43^ ^]^ Patients with TNBC show poorest prognosis among the three breast cancer subtypes. TNBC are highly heterogeneity, and a study claimed that TNBC can be further divided into 16 subtypes.^[^ ^44^ ^]^ To date, druggable discriminators for TNBC are still lacking.^[^ ^45^ ^]^ Here, we reveal that more than 80% TNBC is CLU+, suggesting CLU+ TNBC represents the most common TNBC type. In past decades, a number of studies reported various important regulators in TNBC for development of targeted therapies against TNBC.^[^ ^46^ ^]^ And several targeted therapies, such as Veliparib targeting PARP, Dinaciclib targeting CDKs, and Aftatinib targeting EGFR, have been developed and tested in preclinical and clinical trials.^[^ ^4^, ^47^ ^]^ However, not a targeted therapy is able to show satisfied clinical outcomes, resulting in currently no established clinically targeted therapies for treatment of TNBC. Thus, the effective targeted therapy against TNBC is still an urgent and unmet clinical need. Here, we provided evidences to provide the new molecular mechanism behind the important role of CLU in pathogenesis of TNBC via revealing PRKD3 as a key regulator of CLU. Significantly, we provided in vitro and in vivo preclinical experimental data to show promising effects of targeted therapies against CLU pathway via targeting CLU and PRKD3 for treatment of TNBC. Besides, we also raised sCLU in serum to be a blood‐based biomarker for TNBC.

In conclusion, we define PRKD3 to be a key regulator of CLU in promoting TNBC tumor growth, and provide new promising targeted therapies against CLU pathway via targeting CLU and PRKD3 as well as serum sCLU as a blood‐based biomarker for efficient management of TNBC (Figure 8G).

## 4 Experimental Section

##### Cell Lines and Cell Culture

Human breast cancer cell lines MDA‐MB‐231, MDA‐MB‐468, and 293T were obtained from American type culture collection. All cell lines were cultured in Dulbecco's modified essential medium (DMEM) (Gibco) supplemented with 10% heat‐inactivated fetal bovine serum (Gibco) and 1% penicillin‐streptomycin solution (Gibco) in a humidified incubator with 5% CO_2_ at 37 °C.

##### Clinical Samples

79 tumor tissues from patients with TNBC and blood samples from 24 healthy persons and 37 patients with TNBC were obtained from the First Affiliated Hospital of Soochow University. All patients enrolled in this study were newly diagnosed and had not received any drug treatment. This study was reviewed and approved by the Committee for Ethical Review of Research at the First Affiliated Hospital of Soochow University, and the patients were provided with written informed consent forms. Collection of clinical samples was carried out in accordance with approved guidelines.

##### Patient‐Derived TNBC Organoid Assay

Briefly, a TNBC tissue was digested by Collagenase I, then cultured in DMEM‐F/12 (Gibco) supplemented with 10 mm HEPES (Sigma‐Aldrich), 10 µg mL^−1^ Gentamycin (Euroclone), 2 mm L‐Glutamine (Euroclone), 1% Pennicilin/streptomycin (Euroclone), 2.5 µg mL^−1^ Amphotericin B (Euroclone), 5 mm Nicotinamide (Sigma), 1.25 mm N‐acetylcysteine (Sigma), 1 × B27 supplement (Gibco), 250 ng mL^−1^ R‐spondin 3 (R&D), 5 nm Heregulin (Peprotech), 5 ng mL^−1^ KGF (Peprotech), 20 ng mL^−1^ FGF10 (Peprotech), 5 ng mL^−1^ EGF (Peprotech), 100 ng mL^−1^ Noggin (Peprotech), 500 nm A83–01 (Tocris), 5 µm Y‐27632 (Abmole), 500 nm SB202190 (Sigma). The TNBC organoids were treated with CRT0066101 (1 µm) and/or OGX‐011 (300 nm) for cell growth assay.

##### Real Time RT‐qPCR

Total RNA was extracted using the RNeasy kit (OMEGA). For real‐time RT‐qPCR, cDNAs were synthesized with the PrimeScript RT reagent kit (TaKaRa) and PCR reactions were performed with SYBR Premix Ex Taq (TaKaRa). The primer sequences for RT‐qPCR are listed in Table S3, Supporting Information.

##### Antibodies for Western Blot and Immunofluorescence

The antibodies against HA and FLAG were purchased from Abcam. Antibodies against PRKD3, CLU, and LAMP1 were purchased from Cell Signaling Technology. *β*‐actin antibody was purchased from Sigma. LRP2 antibody was purchased from Absci. Anti‐rabbit, anti‐mouse, and IgG antibodies were purchased from Santa Cruz Biotechnology.

##### Immunoblotting Analysis

For the CHX chase assay, cells were treated with CHX (50 µg mL^−1^) and harvested at the indicated time points. Cells were treated with MG132 (10 µm), CQ (10 µm), or CRT0066101 (1 µm) for 12 h to inhibit UPS, ALP, or PRKDs before harvesting. Treated cells were lysed, and the lysates were analyzed by western blotting.

##### Co‐IP and Silver Staining

Co‐IP was performed using anti‐PRKD3, anti‐CLU antibody (Cell Signaling Technology), anti‐FLAG (Sigma), anti‐HA (Santa Cruz), and Dynabeads Protein G (Invitrogen) according to manufacturer instructions. In brief, cell lysates were incubated with anti‐ antibody‐conjugated beads at 4 °C for 2 h. Then, the beads were washed extensively and boiled in SDS loading buffer. Silver staining, MS analysis, and western blot were used to examine the immunoprecipitated proteins. Silver staining was performed using the Fast Silver Stain Kit (Beyotime) following the protocol provided by manufacturer.

##### Immunofluorescence Staining

TNBC cells were cultured on glass slides in 24‐well plates. PBS was used to wash the cells on glass slides in whole procedure. The cells were fixed with 4% paraformaldehyde (PFA) for more than 30 min at room temperature (RT) and then permeabilized/blocked with PBS containing 0.1% Triton ×‐100/1% BSA for about 1 h at RT. The primary antibodies for PRKD3, CLU, and LAMP1 and the secondary antibodies (Alexa Fluor 555‐conjugated and 488‐conjugated secondary antibody) were used to examine protein distribution in cells. DAPI (Solarbio) was used to label cell nucleus.

##### Cell Proliferation Assay and Colony Formation Assay

CCK‐8 kit (Dojindo Laboratories) was used to measure breast cancer proliferation according to the protocol recommended by manufacturer. Briefly, TNBC cells were plated out at the same confluence in 96‐well dishes and grown at 37 °C for 3 days. CCK‐8 solution was added into each well for 3 h, and then absorbance at 450 nm was measured. For colony formation assay, the cells were trypsinized and incubated in 6‐well plates for 7 days. The solution of 0.1% crystal violet and 20% methanol was used to stain the cell colonies.

##### TNBC Cell Line Xenograft Mouse Model

Female nude mice were purchased from Shanghai Sushang Biology Technology. To generate TNBC tumor in mice, 5 × 10^6^ TNBC cells were injected into the mammary fat pads of the mice. Two weeks later, mice received subcutaneous injections of CRT0066101 (50 mg/kg) and/or OGX‐011 (15 mg/kg) for indicated time periods. Vernier calipers were used to measure tumor width and length weekly, and volume was calculated using the formula 1/2 × (*l*) × (*w*)^2^ [*l*: length; *w*: width].

##### PDX Mouse Model

This method was approved by the medical ethics committee at Suzhou Institute of Biomedical Engineering and Technology. Human TNBC tumor tissues were obtained from the First Affiliated Hospital of Soochow University, and letters of authorization were signed by all the patients who provided tissues. Cells from these tissues were grafted into the mammary fat pad and tumor sizes were measured by the same methods as described in the TNBC cell line xenograft mouse model section.

##### Immunohistochemistry Analysis

Primary breast tumors and PDX xenograft tumors were fixed with 4% PFA, embedded in paraffin blocks, and then micro‐dissected into several thin sections. Sections were then deparaffinized and subjected to antigen retrieval in citric acid buffer (pH 3.5) for 15 min. Sections were then incubated in 1% hydrogen peroxidase for 10 min followed by incubation with HRP‐conjugated antibodies against ER, PR, HER2, PRKD3, or CLU (Cell Signaling Technology) at 4 °C overnight. Staining was performed using the HRP‐IHC kit according to the manufacturer's instructions. The slides were evaluated by two independent pathologists blinded to clinicopathological features and the clinical course, under a light microscope. The staining intensity of PRKD3 or CLU were scored as 0 (negative, −), 1 (weak, +), 2 (moderate, ++), and 3 (strong, +++). The extent of staining was scored as 0–1.0 (0–100%). The final staining score (0–3) was calculated as the multiplication of the intensity score and extent score. A final score of ≥1 was defined as high expression, otherwise scores were defined as low expression.

##### Statistical Analysis

GraphPad Prism 7 software was used for statistical analysis. For statistical analysis, data are presented as the mean ± standard error of mean (SEM) of at least three independent experiments unless otherwise stated, and *t*‐test was used for statistic quantification. “ns”, “*”, “**”, and “***” stand for not significant, *p* <0.05, *p* <0.01, and *p* <0.001, respectively.

## Conflict of Interest

Nanjing Normal University has filed patent applications related to this work, listing L.C., Y.L., and J.Z. as inventors.

## Authors' Contributions

The study was conceived by Y.L. and L.C. designed the experiments. Y.L., Y.Z., and X.M. performed experiments and analyzed data. Y.L. and Y.Z. provided material support. Y.L. and L.C. wrote the manuscript. L.C. critically reviewed the manuscript. All authors read and approved the final manuscript.

## Ethics Approval and Consent to Participate

The study protocols were approved by the Suzhou Institute of Biomedical Engineering and Technology and Nanjing Normal University. The mice were handed via following the Institutional Animal Care and Use Committee guidelines of the Suzhou Institute of Biomedical Engineering and Technology (A‐06).

## Supporting information

Supporting Information

Supplemental Table 1

Supplemental Table 2

Supplemental Table 3

## Data Availability

All data generated or analyzed during this study are included in the manuscript.
