# Supplementary material for: Inhibition Lysosomal Degradation of Clusterin by Protein Kinase D3 Promotes Triple‐Negative Breast Cancer Tumor Growth
Source: Adv Sci (Weinh). 2021 Jan 6;8(4):2003205. doi: 10.1002/advs.202003205 (PMC7887572; doi:10.1002/advs.202003205)
Supplement: Supplementary file 1 — Supporting Information [file ADVS-8-2003205-s001.pdf]

© 2020 Wiley-VCH GmbH

ADVANCED SCIENCE
Open Access

Supporting Information

for *Adv. Sci.,* DOI: 10.1002/advs.202003205

Inhibition lysosomal degradation of Clustrin by Protein Kinase D3 promotes triple-negative breast cancer tumor growth

Yan Liu[1,2], Yehui Zhou[3], Xinxing Ma[3], Liming Chen[2]*

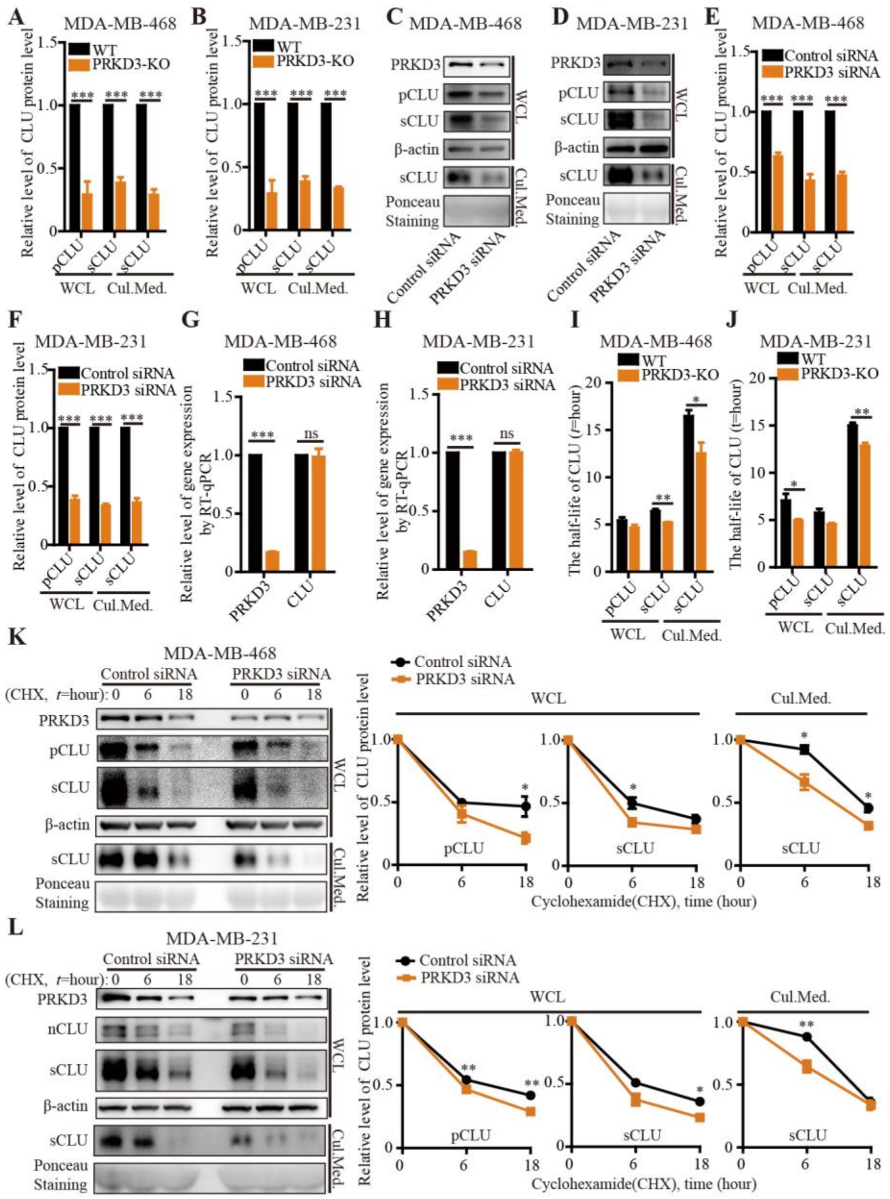

Figure S1 related to Figure 2.

(A) Densitometric analysis of western blot in Figure 2A. **(B)** Densitometric analysis of western blot in Figure 2B. **(C-D)** Representative western blot show that the knock-down PRKD3 by siRNA decease the protein level in MDA-MB-468 (C) and MDA-MB-231 (D). **(E)** Densitometric analysis of western blot in (C). **(F)** Densitometric analysis of western blot in (D). **(G-H)** RT-qPCR results show that the knock-down PRKD3 by siRNA didn't decease the mRNA level of *CLU* in MDA-MB-468 (G) and MDA-MB-231 (H). **(I)** Estimated half-life of CLU in Figure 2E. **(J)** Estimated half-life of CLU in Figure 2F. **(K-L)** Representative western blots for cycloheximide (CHX)-chasing assays show that knock-down PRKD3 by siRNA decreases of protein

stabilities of pCLU and sCLU in cells as well as sCLU in cell culture mediums in two TNBC cell lines compared to control siRNA: MDA-MB-468 (K) and MDA-MB-231 (L). The error bars represent the mean ± SEM. Statistics analyses were performed using t-test: *$p < 0.05$, **$p < 0.01$, and ***$p < 0.001$.

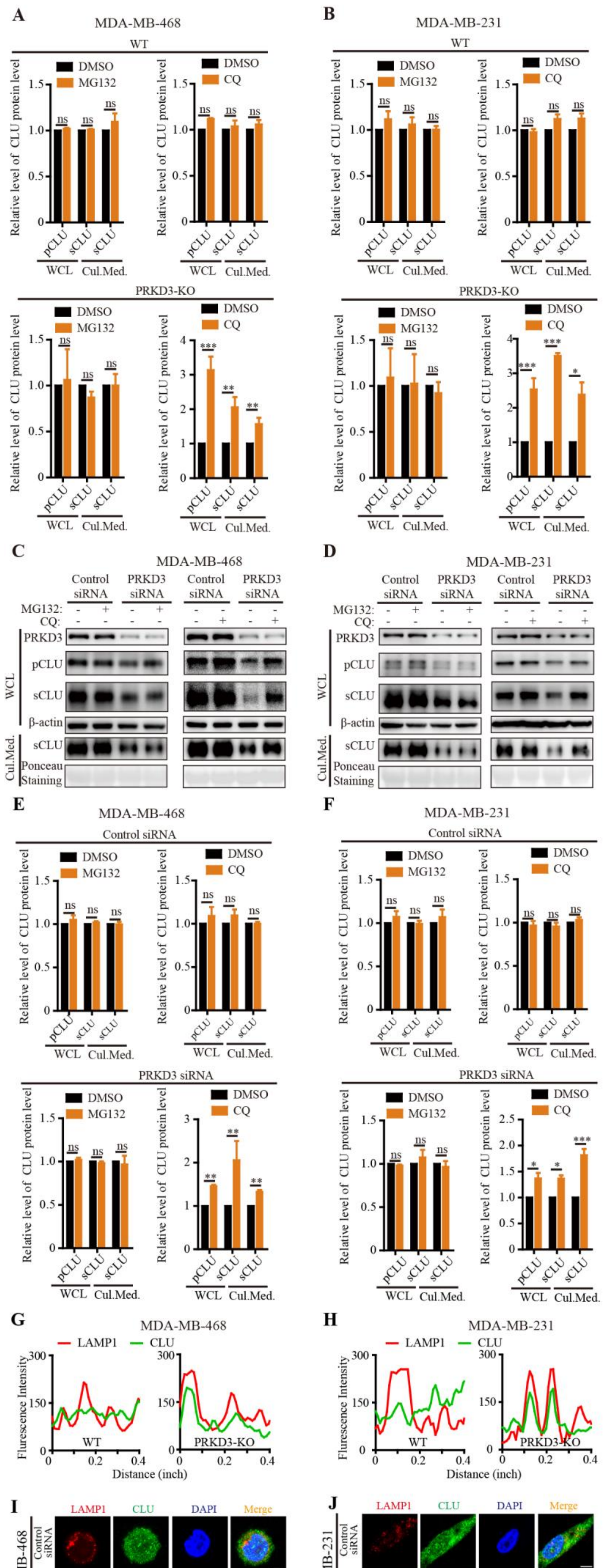
A
MDA-MB-468
WT
PRKD3-KO
B
MDA-MB-231
DMSO
MG132
CQ
Relative level of CLU protein level
pCLU
sCLU
WCL
Cul.Med.
ns
C
D
Control siRNA
PRKD3 siRNA
MG132:
CQ:
PRKD3
β-actin
Ponceau Staining
E
F
G
H
LAMP1
CLU
Flurescence Intensity
Distance (inch)
I
J
DAPI
Merge

Figure S2 related to Figure 3.

(A) Densitometric analysis of western blot in Figure 3A. **(B)** Densitometric analysis of western blot in Figure 3B. **(C-D)** Representative western blots show that treatment of lysosomal degradation inhibitor-chloroquine (CQ) but not proteasomal degradation inhibitor-MG132 can increase protein levels of pCLU and sCLU in cells as well as sCLU in cell culture medium upon PRKD3 knocked down by siRNA in two TNBC cell lines compared to control: MDA-MB-468 (C) and MDA-MB-231 (D). **(E)** Densitometric analysis of western blot in (C). **(F)** Densitometric analysis of western blot (D). **(G)** Colocalization analysis of Figure 3C. **(H)** Colocalization analysis of Figure 3D. **(I-J)** Representative confocal images from immunofluorescent assays using LAMP1 as the lysosome marker consistently show that there are obvious increases of CLU-LAMP1 colocalization foci upon PRKD3 knocked down by siRNA in two TNBC cell lines compared to control: MDA-MB-468 (I) and MDA-MB-231 (J). Several representative CLU-LAMP1 colocalization foci were indicated by arrows. Sale bar indicates 2.5 μm. **(K)** Colocalization analysis of (I). **(L)** Colocalization analysis of (J). **(M-N)** Representative western blots of Co-IP assays show that the interaction of endogenous CLU and LRP2 is increased in two PRKD3-KO TNBC cell lines compared to WT cells: MDA-MB-468 (M) and MDA-MB-231 (N). **(O)** Densitometric analysis of western blot in (M). **(P)** Densitometric analysis of western blot in (N). The error bars represent the mean ± SEM. t-test; *p < 0.05, **p < 0.01, and ***p < 0.001.

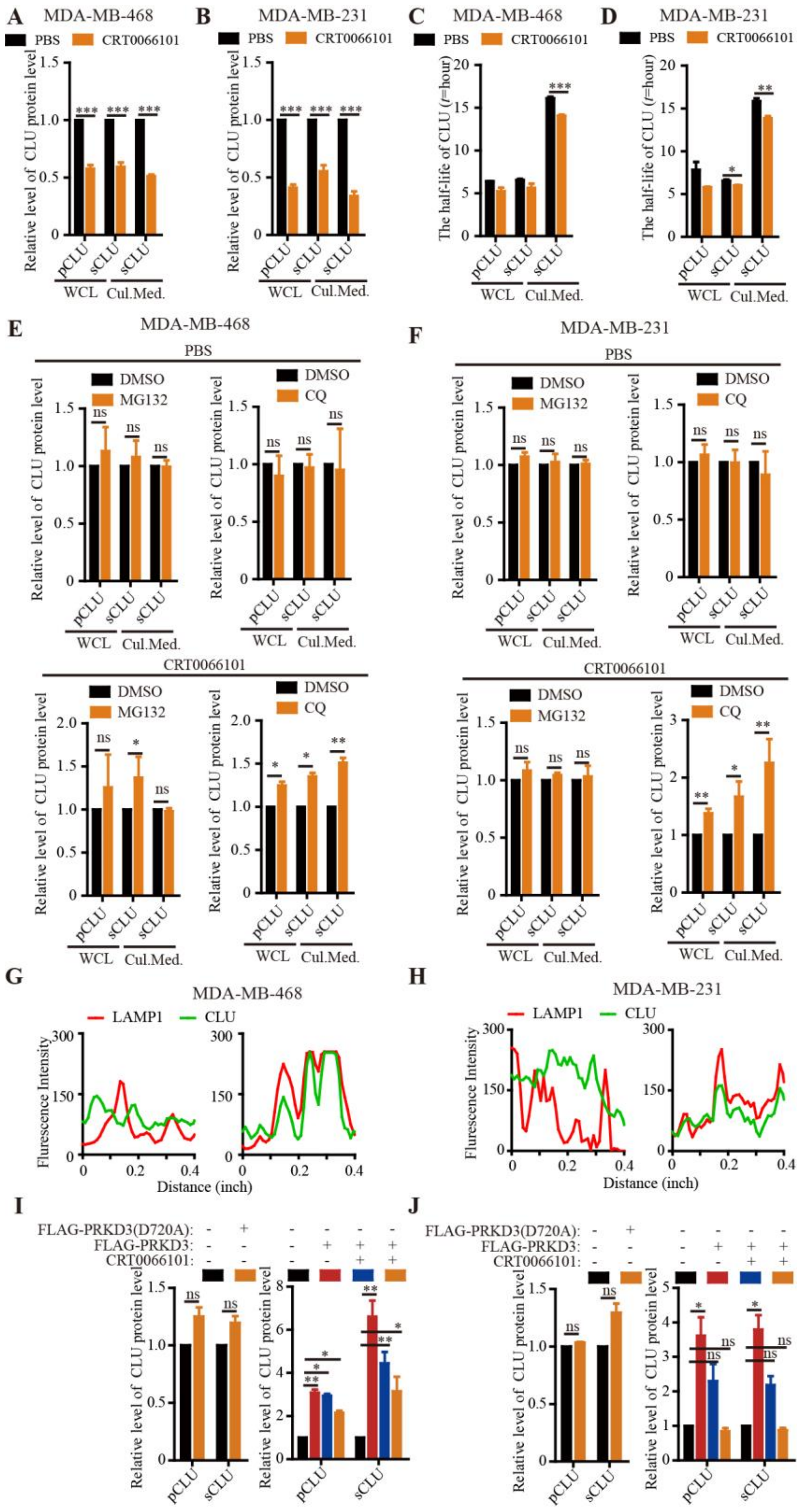

Figure S3-related to Figure 4.

(A) Densitometric analysis of western blot in Figure 4C. **(B)** Densitometric analysis of western blot in Figure 4D. **(C)** Estimated half-life of CLU in Figure 4E. **(D)** Estimated half-life of CLU in Figure 4F. **(E)** Densitometric analysis of western blot in Figure

4G. **(F)** Densitometric analysis of western blot in Figure 4H. **(G)** Colocalization analysis of Figure 4I. **(H)** Colocalization analysis of Figure 4J. **(I)** Densitometric analysis of western blot in Figure 4K. **(J)** Densitometric analysis of western blot in Figure 4L. The error bars represent the mean ± SEM. The error bars represent the mean ± SEM. t-test; $*p < 0.05$, $**p < 0.01$, and $***p < 0.001$.

Table S1. PRKD3 binding proteins identified by Mass Spectrometry (MS)

Table S2. The clinical information of tumor samples from patients with TNBC.

Table S3. Primer sequences for RT-qPCR
